# Radiomic Analysis of CT Predicts Tumor Response in Human Lung Cancer with Radiotherapy

**DOI:** 10.1007/s10278-020-00385-3

**Published:** 2020-10-06

**Authors:** Mengmeng Yan, Weidong Wang

**Affiliations:** 1 Urban Vocational College of Sichuan, Chengdu, China; 2 Sichuan Cancer Hospital & Institute, Chengdu, China; 3 Department of Radiation Oncology, Sichuan Cancer Hospital & Institute, Chengdu, 610041 China; 4 Radiation Oncology, Key Laboratory of Sichuan Province, Chengdu, China

**Keywords:** Radiomics, Lung cancer, Radiotherapy, Machine learning

## Abstract

**Purpose:**

Radiomics features can be positioned to monitor changes throughout treatment. In this study, we evaluated machine learning for predicting tumor response by analyzing CT images of lung cancer patients treated with radiotherapy.

**Experimental Design:**

For this retrospective study, screening or standard diagnostic CT images were collected for 100 patients (mean age, 67 years; range, 55–82 years; 64 men [mean age, 68 years; range, 55–82 years] and 36 women [mean age, 65 years; range, 60–72 years]) from two institutions between 2013 and 2017. Radiomics analysis was available for each patient. Features were pruned to train machine learning classifiers with 50 patients, then trained in the test dataset.

**Result:**

A support vector machine classifier with 2 radiomic features (flatness and coefficient of variation) achieved an area under the receiver operating characteristic curve (AUC) of 0.91 on the test set.

**Conclusion:**

The 2 radiomic features, flatness, and coefficient of variation, from the volume of interest of lung tumor, can be the biomarkers for predicting tumor response at CT.

## Introduction

Radiation therapy is a crucial and cost-effective lung cancer curative treatment [1], and its curative effect largely depends on the radiosensitivity of tumor cells of the different patients [2]. So, the valuation of radiosensitivity with respect to radiotherapy has significant potential to contribute to further therapeutic gain.

Radiomics provides a quantitative method to mine useful data as much as possible from medical images and can be applied to clinical decision support systems [3–10]. And CT-based radiomics can quantify tumor phenotypic differences in CT images using radiomic features. Radiomic features (such as intensity, shape, texture, or wavelet), extracted from medical images, when combined with clinical parameters can make clinical decision more precise [11]. It has shown a great ability to be the biomarkers in predicting clinical events of lung cancer patients, recent examples like predicting the response of enzymes, gene and immunity therapy which are associated with lung tumor [12], evaluating the drug reaction [13], radiation pneumonitis [14], and distinguishing lung cancer histologic subtypes [15].

However, there are not such researches about the radiosensitivity of human lung cancer with CT-based radiomics until now. And radiotherapy dose still needs to be more precise.

This paper tests the hypothesis that radiomic features have a mathematical relationship with tumor response, which can be predicted by a proper model of radiomics. To invest the evidence of that hypothesis, we extracted quantitative image features from CT images and made a radiomics analysis.

## Materials and Methods

### Patients and Datasets

Our work was approved by the institutional ethics committee.

Our work incorporates VOI radiomics evaluates a machine learning approach and predicts tumor response. We collected retrospectively three independent radiotherapy datasets for this work, one training dataset comes from our institute and one testing dataset comes from Chengdu hospital.

The training dataset consists of 50 NSCLC stage II–IV patients, 30 men (mean age, 67 years; range, 55–80 years) and 20 women (mean age, 68 years; range, 55–72 years), imaged with CT, with or without intravenous contrast, and treated with radiation therapy at another institute in Chengdu, China. Images were acquired between 2010 and 2017. This data set was used for feature selection.

The testing dataset consists of 50 NSCLC stage II–IV patients, 34 men (mean age, 62 years; range, 58–78 years) and 16 women (mean age, 64 years; range, 55–80 years), imaged with CT, with or without intravenous contrast, and treated with radiation therapy at our institute. Images were acquired between 2010 and 2017. This data set was used for model building.

CT images for each patient include two parts: the CT before and after radiotherapy. The CT before radiotherapy was collected from radiotherapy positioning images, the CT after radiotherapy collected from the CT whose scan time CT scan time within 1 to 3 months after radiation therapy.

### Tumor Segment and Feature Extraction

The volume of interest was manually delineated by two thoracic radiologists (with 20 years and 11 years of experience in lung CT). And the influence of CT scan protocol (manufacturer, section thickness, type of image) was reduced by the combat method [16].

Radiomics features were extracted automatically with CERR software [17]. All feature values were normalized (mean of 0 and a standard deviation of 1).

### Statistical Analysis

The statistical analysis reported in our study was performed with Matlab (Mathworks). The reliefF algorithm and Fast Correlation Based Filter (FCBF) algorithm were implemented as a feature selection method. reliefF was used to calculate the ability of each feature to distinguish between classes on similar data instances. And FCBF is used to discriminate between redundant features. To avoid the curse of dimensionality and reduce the risk of overfitting, we compared the prediction ability of each feature by classifiers, then only the top 2 features were used for further analysis.

To explore the association of the radiomics features with tumor response, top 2 features were used to train multiple classifiers (support vector machine (SVM), adaptive boosting, random forest, decision tree) and validated on this data set.

Tumor response scores (score 0 was “progressive disease” or “stable disease”, score 1 was “partial response” or “complete response”) were set for the target of the radiomics model.

## Result

### Radiomics Features from the VOI Describes Tumor Response

The top 2 radiomics features obtained from the training set are flatness (belongs to morphological features) that describes the geometric length aspect of VOI, and coefficient of variation (belongs to intensity-based statistical features) that describes a continuous intensity distribution.

### Radiomics Model

The top 2 features were applied to the test data set with multiple classifiers and validated in the data set using multiple indexes (area under ROC, classification accuracy, F-1, precision, and recall). Given the small data set, we sampled the test data set with leave-one-out. It holds out one instance at a time, inducing the model from all others and then classifying the held out instances. This method is obviously very stable and reliable.

The highest AUC on the test set of 0.912 was obtained by using the SVM classifier with a linear kernel. The other performance metrics were accuracy of 88%, F-1 of 88%, precision of 88%, and recall of 63%. Table 1 lists the performance metrics of the classifiers.

**Table 1:** The performance metrics of the classifiers

Method	AUC	CA	F1	Precision	Recall
SVM	0.912	0.880	0.880	0.882	0.880
AdaBoost	0.880	0.880	0.880	0.880	0.880
Random Forest	0.862	0.900	0.900	0.901	0.900
Tree	0.813	0.840	0.840	0.842	0.840

*SVM*, support vector machine; *AdaBoost*, adaptive boosting

## Discussion

In this study, we investigated the ability of radiomic features extracted from lung tumors on CT images to predict tumor response after radiotherapy.

We found that lower-order features have a better predictive ability than higher-order inside the tumor. The radiomics feature of flatness comes from morphological features and coefficient of variation comes from intensity-based statistical features that have the best predictive ability on training data set. And with these top 2 features, SVM classifier had a good performance (AUC of 91%, accuracy of 88%, and precision of 88%).

Our findings are in consensus with Xu et al. [18], who reported that radiomics biomarkers can have a significant impact in predicting treatment response given their low cost and minimal requirements for human input.

There are a genome-based model for adjusting radiotherapy dose (GARD) [19] combined radiosensitivity index and the linear quadratic model, which was reported to predict the tumor response and proper radiotherapy dose. Applying GARD to clinical decision support systems remains to be defined, and the cost of genetic testing is expensive.

The retrospective design of our cohort was restricted to only lung cancer and short of datasets. Further work is needed to focus on enlarging the dataset and make prediction outcomes more precision.

In conclusion, we introduced a machine learning approach that predicts the tumor response of lung cancer patients who are treated with radiotherapy. The radiomics features, flatness, and coefficient of variation had a good performance on the machine learning classifiers.
